# Acetate of Lead, in Cholera and Dysentery

**Published:** 1836

**Authors:** A. B. Price

**Affiliations:** Montgomery county, Centreville, Ohio


### Art. II.—Acetate of Lead in Cholera and Dysentery.

*Observations on the use of Sugar of Lead in the treatment of Cholera and Dysentery.* By Dr. A. B. Price, of Montgomery county, Ohio.

In the Western Journal of the Medical and Physical Sciences, for October, November and December, 1834, page 402, is an article by the Editor, on the use of acetate of lead in epidemic cholera. From the favorable report made of its effects, I determined to give it a trial the first opportunity that offered.

In June, 1835, I was called to a case, the first since reading the article above alluded to. A young man, after having a slight serous diarrhœa, for two or three days, was attacked suddenly with vomiting and profuse rice-water discharges, attended with loss of heat in the extremities; general shrinking of the system, and suspension of the pulse at the wrist; intense thirst, and a burning sensation in the stomach; breathing slow; tongue cool, and palid; heat of the body higher than natural; entire suspension of urinary discharge. In this condition I found him, three hours after the first paroxysm of vomiting; had vomited only three times. I made use of all the means generally resorted to in this desperate disease, in addition to the sugar of lead, which was largely given, yet the patient died on the 4th day. The prescription made use of, was 10 grains of acet. plumb. and 1 of opium; which entirely checked the vomiting and purging. Bilious discharges were procured by calomel, rheubarb and aloes, but the system never recovered from the shock it received at first. Such was the result in another member of the same family, though the lead and opium effectually constipated the bowels, and arrested the vomiting: and in due time bilious discharges were procured by cathartic medicines; yet the patient died, without ever regaining the lost heat of the extremities, although all the external means calculated to effect this object, were used with the most persevering diligence. That the secretion of bile will return, if that of serum be arrested, is established by the above facts: but that health will follow the restoration of the discharge of bile, is equally established not to be a fact; hence the return of bilious stools must not be always hailed as the harbinger of final success.

In both of these cases, purgation was with difficulty brought about, after the use of the lead and opium; and in one case was only effected by croton oil, after the use of a variety of cathartics to no effect. Another case occurred in July, in which the lead was used daily for the suppression of serous diarrhœa, but with partial success, owing no doubt to the patient refusing to remain within doors and neglecting to continue it long enough. After checking the diarrhœa twice or thrice, it was by neglect discontinued, until the disease broke forth with increased energy, when it was again had recourse to, but without the same good effects as before; for under its use the disease continued to progress until a dose of calomel of forty grains was given, after which the discharges ceased. This was repeated three times, when dark bilious fœtid stools appeared. This patient had consecutive fever, for which he was freely bled once, with manifest advantage.

In this case a fair use of the lead was made, after the severe symptoms set in. It was given in large doses, and often repeated, but without allaying the irritability of the stomach or suspending the rice-water discharges.

The above cases stand in opposition, in one respect, to those related in the article alluded to, to wit: that in those, bilious discharges speedily followed the cessation of the serous evacuations, without the use of any other medicine for that purpose. Such was not the fact in these cases; for it required the most powerful cathartics in one case to unloose the bowels, while in others bilious discharges only were produced by calomel.

In mild cases of this disease, and when early seen, the sugar of lead I have no doubt, from what I have seen of its effects and the reports of others, is a remedy of great value; but in the advanced stage, or in those of a severe character at the onset, it will, like all other means which have been so confidently offered to the profession, often fail.

So much for my experience in this matter. I shall now give a more weighty authority, that of Dr. Graves, of Dublin, whose opinions should not be neglected. He claims the credit of first using and recommending it in this disease. He says, "I believe I can fairly claim the merit, such as it is, of being the first to give it in large and effectual doses." His large doses would be considered small ones in this country. Here is his formula; acetate of lead one scruple, opium one grain made into twelve pills, one every half hour, until the rice water discharges from the stomach and bowels begin to diminish. He says that "in all cases where medicine promised any chance of relief, this remedy was attended with the very best of effects; it gradually checked the serous discharge from the bowels, and stopped the vomiting." The importance of this remedy will readily be granted, when it is considered that, unless the discharge of serum be checked, there is no hope of recovery—for it is well known, that so long as the rice water discharges continue, the disease progresses, and that when we succeed in allaying the vomiting and suspend the purging, we have some hopes of being able to master the disease. He recommends that the pills be gradually discontinued. In this way he has given as much as forty grains in twenty-four hours, with great advantage to the patient, and without any bad consequences ensuing. Such has been the success of this practice in Dublin, the doctor observes, that it has become almost universal, and almost superseded the use of calomel and opium. Here is sufficient evidence to justify the use of this article in this terrible disease—and if found to succeed as represented, cholera will be stripped of half its terrors, and the world freed from a horrible scourge.

We propose now to say something of its powers over other affections of the alimentary canal, especially dysentery. In this disease I consider it an invaluable remedy, producing effects that we cannot obtain from any other article of the materia medica. It is highly recommended by many writers who have tried it in this complaint. Dr. Chapman says, "evacuations having been premised, by venesection and purging, I have found nothing more effectually to relieve tormina and tenesmus, to correct the morbid secretions of the intestinal canal, to allay febrile excitement, or to conduce to the comfort and general improvement of the condition of the patient." This entirely accords with my experience with this article, although I think venesection and purging are not always required. I have used it in many cases at the onset of the disease, and after it had been some time standing, and have had no reason to be dissatisfied with the result. It is conceded by all, that dysentery consists in an inflammation of the mucous coat of the intestines, and is principally confined to the large ones. When the inflammation is extensive and intense, attended with much excitement, great pain and abdominal soreness, the antiphlogistic regimen is undoubtedly advisable. But there are many cases in which the attack is slight, yet the stools are frequent, mucous and bloody, attended with tormina and tenesmus, &c. with but little pain, and tenderness of the abdomen. In such cases the sugar of lead may be used, without bleeding or purging being premised.

There seems to be in this disease, as in cholera, a suspension of the secretion of bile; at least this has been the case in all the subjects of the disease with which I have met—hence I have constantly united calomel with the acetate, with great advantage; for when I have been able to procure bilious stools, the patient has recovered. To confirm what is above stated, I will add a few cases from my note book.

Aug. 29, 1835. Was called to see Mrs. V. who was re-presented to be in a dying situation. Under the direction of a *steam doctor*, she had taken great quantities of lobelia inflata, cayenne pepper, and other stimulants. I found her in extreme pain, with such great abdominal soreness that she could scarcely bear the pressure of the bed-clothes, evacuations bloody and mucous, great tormina and tenesmus, pulse frequent and small, great nausea, extremities cool, tongue furred, brown on the sides, red at the tip and edges, extreme thirst, countenance indicative of great agony. I left her the following prescription: opium 1 gr. cal. 3 gr. acet. plumbi. 3 gr., to be taken every hour until she gets relief from pain, and then every two hours afterwards. Ordered injections of acet. plumb. and laudanum, mustard sinapisms to the abdomen.

Aug. 30. Visited her again. Reports that the pain considerably abated after the first dose, frequency of the discharge diminished, less soreness of the abdomen, countenance more pleasant, stools dark and fœtid, containing lumps of a whitish substance like pus, less blood. Medicine continued, with the addition of half gr. ipecac., mucilage of gum arabic for drink, flannel dipped in hot spirits to the abdomen.

31. Has had no discharge for the last twenty-four hours. Skin warm, tongue cleaner, bitter taste in the mouth, and occasionally throws up a mouthful of a green bitter fluid. Cathartic of sulphas. mag. and after its operation the following every four hours: Cal. five gr. opii. one, ipeca. one-half—fomentations continued.

Sept. 1. Salts operated well, bringing bilious stools; less soreness of the abdomen; calls to stool rather more frequent, and more of a dysenteric character; more pain and tenesmus; mouth sore; saliva flowing freely; pulse much excited. Ordered sugar of lead in the place of the calomel, once in four hours; fomentations and medicines continued.

Sept. 2. Condition improved, medicine continued.

Sept. 3. Improving. Left Dover's powder and lead, once in six hours, 5 gr. blue pill at night, decoction of dewberry; convalesced from this on; recovery rapid.

This was a violent case, for when I first saw her, she had been sick only three days. The good effects of the lead was manifest, for on the third day after she commenced the use of it, it was discontinued, and the frequency of the motions were increased, together with more pain, soreness and tenesmus—which symptoms were again soon palliated on resuming it. In five days she took near 160 grains, without any inconvenience arising from it.

Case II. was attacked with the ordinary symptoms of dysentery, for which a variety of domestic remedies were used without any advantage, before I was called. When I first saw her, she had slight fever, with severe pain in the abdomen, stools frequent and containing much blood, tongue brown. Bled her and gave calomel 20 gr. Dov. pow. 10, to be followed by castor oil. After the operation of the medicine she took a dose of laudanum. Next day, no improvement; the usual means were resorted to in this case, but without much benefit. On the fifth day from the time I first saw her, I gave her sugar of lead in four gr. doses, combined with one gr. of opium, and one-half grain of ipecac. from which time she gradually convalesced.

Many other cases might be mentioned, but these must suffice. Dysentery prevailed to a considerable extent east of this place during the summer of 1835, and in all of the cases that came under my care, (and they were not a few,) I used this article, and with increased confidence in its remediate powers in this disease.

It seems to me, that the acetate of lead, in combination with opium where there is much pain, with ipecac. where there is dryness of the skin and fever, and with calomel when biliary derangement is present, answers every indication of the disease; for it is laid down by our best authors, and is the practice of most of our physicians, to moderate general excitement when excessive—to correct hepatic derangement to restore the disordered functions of the skin—and particularly to allay local symptoms; for all which the above combination is admirably adapted.

It is an article every way worthy of the confidence of the profession; but from a mistaken notion of its deleterious qualities, when used internally, it has been resorted to with a sparing hand, and with the greatest apprehensions. But those unfounded notions are gradually being dispelled by the light of experience, and ere long it will be used as fearlessly as any other article of the materia medica. It cannot be denied that the internal administration of the acetate of lead has been occasionally detrimental, but this should not deter us from using at all—as it is known that all of our active remedies are, when given without proper guards, or under unfavorable circumstances, deadly in their effects. Yet who would forsake the use of them on this ground?

I have used it in many cases, and continued it from one day to three weeks, without experiencing any bad effects from it.

I will here introduce, for the benefit of those who may still entertain fears respecting the internal use of this article, the evidence of Dr. Stokes, of London, a name familiar to the readers of the Western Journal. He says, "hitherto many persons have been afraid to employ it in large quantities, from the fear of producing painter's colic; but at present it is known that this disease is to be attributed to the absorption of the carbonate of lead in almost every instance, and that the acetate is comparatively harmless. On this point I can mention one interesting fact, namely, that I have been in the constant habit of using it, and in considerable doses, for the last six years, and I cannot bring to my recollection one single instance of colic produced by it. One patient, in particular, who was under my care, took it in considerable doses for six weeks, and without any apparent injury. The only cases, in which I have seen the acetate act as a poison, were in those in which it had been used as an external application."

In order the more effectually to prevent an accumulation of this article in the system, I have given, every second, third or fourth day, as necessity seemed to require, a cathartic of oil or sulph. mag. This course is necessary to the final cure. I would prefer the saline cathartic to any other—for the simple reason, that I have found it the most beneficial. This, perhaps, is owing to its stimulating effects on the intestinal exhalents. Indeed they act as depletants, without producing as much griping as oil or rheubarb; and hence it is reasonable to suppose they would be more beneficial.

A. B. PRICE.

*Centreville, O., March* 29.

